# Infection and exposure to vector-borne pathogens in rural dogs and their ticks, Uganda

**DOI:** 10.1186/s13071-015-0919-x

**Published:** 2015-06-05

**Authors:** Tatiana Proboste, Gladys Kalema-Zikusoka, Laura Altet, Laia Solano-Gallego, Isabel G. Fernández de Mera, Andrea D. Chirife, Jesús Muro, Ester Bach, Antonio Piazza, Aitor Cevidanes, Valeria Blanda, Lawrence Mugisha, José de la Fuente, Santo Caracappa, Javier Millán

**Affiliations:** Facultad de Ciencias Silvoagropecuarias, Universidad Mayor, Santiago, Chile; Conservation Through Public Health, Plot 3 Mapera Lane, Uringi Crescent, Entebbe, Uganda; VetGenomics, Autonomous University of Barcelona, Cerdanyola, 08193 Barcelona Spain; Departament de Medicina i Cirurgia Animals, Universitat Autònoma de Barcelona, Cerdanyola, 08193 Barcelona Spain; Health & Biotechnology (SaBio) Group, IREC (CSIC-UCLM- JCCM), Ronda de Toledo s/n, Ciudad Real, 13005 Spain; Camino del Rosario P5, Peñaflor, Santiago Chile; Andorra Veterinary Services, Government of Andorra, Andorra La Vella, Andorra; Servei d’Hematologia Clinica Veterinaria (SHCV), Departament de Medicina i Cirurgia Animals, Facultat de Veterinaria, Universitat Autònoma de Barcelona, Cerdanyola, 08193 Barcelona Spain; Istituto Zooprofilattico Sperimentale della Sicilia, via Gino Marinuzzi 3, 90129 Palermo, Italy; Facultad de Ecología y Recursos Naturales, Universidad Andres Bello, Republica 252, Santiago, Chile; College of Veterinary Medicine, Animal Resources and Biosecurity, Makerere University, Kampala, 7062 Uganda; Conservation & Ecosystem Health Alliance (CEHA), Kampala, 34153 Uganda; Department of Veterinary Pathobiology, Center for Veterinary Health Sciences, Oklahoma State University, Stillwater, 74078 OK USA

**Keywords:** *Babesia*, Dogs, East Africa, *Ehrlichia*, *Rickettsia*, Tick-borne pathogens

## Abstract

**Background:**

In rural parts of Africa, dogs live in close association with humans and livestock, roam freely, and usually do not receive prophylactic measures. Thus, they are a source of infectious disease for humans and for wildlife such as protected carnivores. In 2011, an epidemiological study was carried out around three conservation areas in Uganda to detect the presence and determine the prevalence of vector-borne pathogens in rural dogs and associated ticks to evaluate the risk that these pathogens pose to humans and wildlife.

**Methods:**

Serum samples (*n* = 105), blood smears (*n* = 43) and blood preserved on FTA cards (*n* = 38) and ticks (58 monospecific pools of *Haemaphysalis leachi* and *Rhipicephalus praetextatus* including 312 ticks from 52 dogs) were collected from dogs. Dog sera were tested by indirect immunofluorescence to detect the presence of antibodies against *Rickettsia conorii* and *Ehrlichia canis*. Antibodies against *R. conorii* were also examined by indirect enzyme immunoassay. Real time PCR for the detection of *Rickettsia* spp., Anaplasmataceae, *Bartonella* spp. and *Babesia* spp. was performed in DNA extracted from FTA cards and ticks.

**Results:**

99 % of the dogs were seropositive to *Rickettsia* spp. and 29.5 % to *Ehrlichia* spp. Molecular analyses revealed that 7.8 % of the blood samples were infected with *Babesia rossi*, and all were negative for *Rickettsia* spp. and *Ehrlichia* spp. Ticks were infected with *Rickettsia* sp. (18.9 %), including *R. conorii* and *R. massiliae*; *Ehrlichia* sp. (18.9 %), including *E. chaffeensis* and *Anaplasma platys*; and *B. rossi* (1.7 %). *Bartonella* spp. was not detected in any of the blood or tick samples.

**Conclusions:**

This study confirms the presence of previously undetected vector-borne pathogens of humans and animals in East Africa. We recommend that dog owners in rural Uganda be advised to protect their animals against ectoparasites to prevent the transmission of pathogens to humans and wildlife.

## Background

Domestic dogs live in close association with humans and livestock. At a global scale, one of the main implications of this relationship is the transmission of zoonotic diseases [1], with dogs participating in the transmission of over 60 zoonoses [2]. Traditionally, households in Africa keep dogs for hunting, herding, security, and guarding livestock and scaring off vermin in nearby protected areas [3]. In Uganda, like elsewhere in East Africa, most rural dogs roam freely. This behavior exposes them to pathogens from consuming garbage, rodents and carcasses and through inhalation during scent communication. In addition, dogs receive no prophylactic measures such as vaccinations. Indeed, a recent study demonstrated a high seroprevalence to important human and animal pathogens in Ugandan dogs, including rabies virus, canine distemper virus, parvovirus, *Leishmania donovani* and *Toxoplasma gondii* [4]. The principal routes of transmission of zoonotic infection from dogs to humans are bites, ingestion of fecal material and arthropod vectors [1]. On the other hand, ticks are the most important vectors of disease-causing pathogens in domestic and wild animals [5]. Tick-transmitted infections are an emerging problem in dogs and have recently become a major focus of interest in areas of the world in which they have traditionally been considered non-endemic. This relates to both their significance to canine health, and to the possible reservoir status of the dog of potentially zoonotic disease [6]. In addition, untreated animals like the rural dogs studied here can serve as sentinels for tick infestation in the environment and for pathogen diversity in the tick population, and pathogen incidence in the dog population can reflect pathogen infection pressure [7].

Dogs are carriers of tick-borne rickettsioses, which are important emerging vector-borne infections of humans worldwide, including in sub-Saharan Africa [8–10]. Six tick-borne spotted fever group pathogenic rickettsiae are known to occur in sub-Saharan Africa: *Rickettsia conorii conorii*, the agent of Mediterranean spotted fever; *R. c. caspia,* the agent of Astrakhan fever; *R. africae*, the agent of African tick-bite fever; and *R. aeschlimannii*, *R. sibirica mongolitimonae* and *R. massiliae* [10]. In Uganda, Socolovschi *et al.* [11] detected *R. conorii* in *Haemaphysalis punctaleachi* ticks collected from a dog in Kampala. This pathogen was also detected in *H. leachi* in Zimbabwe [12]. Dogs can also be infected by members of Anaplasmataceae, which are rickettsial organisms that infect human and animal leukocytes [13]. Agents such as *Ehrlichia chaffeensis* and *E. ewingii* cause human infections of varying severity, and are considered to be emerging tick-borne zoonoses [14]. In Cameroon, *E. chaffeensis* was detected in ticks from dogs in one kennel [15] and sequences similar to *E. chaffeensis, E. canis* and *E. ewingii* were detected in ticks from Mali and Niger [16].

Other vector-borne bacteria with potentially serious clinical implications are those belonging to the genus *Bartonella* [17]. At present, more than 20 species or subspecies of *Bartonella* have been described and 12 of these are recognized as zoonotic human pathogens [18]. *Bartonella* spp. has been detected in different mammal species in sub-Saharan Africa. In Nigeria, high prevalence of infection with *Bartonella* spp. was reported in commensal rodents and associated ectoparasites (ticks, fleas and mites), whereas in Zimbabwe, *B. henselae* was isolated from a captive cheetah [19, 20]. Finally, parasites belonging to the genus *Babesia* are protozoa found in domestic animals and are transmitted by ticks. Babesiosis is particularly severe in naïve animals introduced into endemic areas. In Africa, epidemiological studies of canine babesiosis using molecular methods have been carried out only in South Africa, Sudan and Nigeria, where *B. rossi* and *B. vogeli* were shown to be present [21–23].

The aim of this work was to detect exposure to selected vector-borne pathogens in rural dogs and associated ticks in Uganda, determine their prevalence, and characterize the implicated pathogens using molecular methods.

## Methods

### Study area

**Fig. 1:**
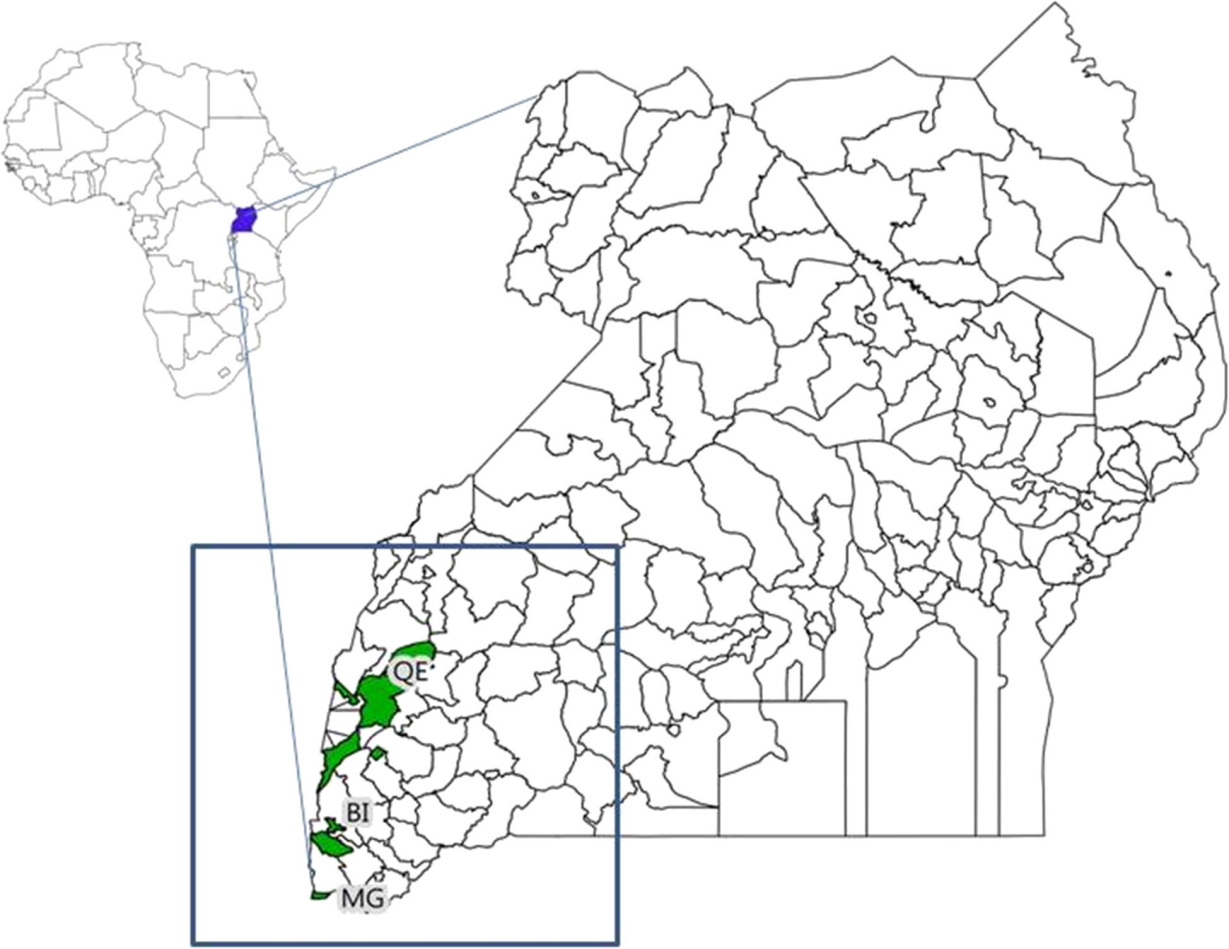
National Map of Uganda, showing the three study areas: (QE) Queen Elizabeth National Park, (BI) Bwindi Impenetrable National Park, (MG) Mgahinga Gorilla Park

Dogs were sampled in 2011 during a rabies vaccination campaign in and near three conservation areas in southwestern Uganda: Bwindi Impenetrable National Park (BI), Mgahinga Gorilla National Park (MG), and Queen Elizabeth National Park (QE) (Fig. 1). BI and MG are located on the rim of the Rift Valley. These two parks host some of the most biologically diverse tropical forest in East Africa and are home to more than half of the world’s remaining mountain gorillas (*Gorilla beringei beringei*). QE includes a diversity of habitats, including savannah, wetlands and lowland forests, and is home to populations of protected carnivores and ungulates. These parks lie within a densely populated rural landscape; in some areas, the human population is as high as 500 people/km^2^, and is highest around BI. This has led to high levels of interaction between local communities and their domestic animals and local wildlife [4].

### Sampling

Two hundred and fifty-one dogs were sampled for when voluntarily brought in by their owners. Blood was obtained from the cephalic vein of 105 dogs: 91 dogs were adults (more than 12 months old) and 14 were young (between 6 and 12 months old). Blood was collected in serum separator tubes and allowed to clot, and then centrifuged at 50 g for 15 min. The serum was removed and cryopreserved in liquid nitrogen until arrival at the laboratory, where it was frozen at −20 °C. Thirty-eight blood samples were applied (100 μl) to FTA™ Nucleic Acid Collection Cards (Whatman, Maidstone, Kent, UK), air dried, and stored in sealed plastic bags until further processing.

A total of 430 ticks were collected from 101 dogs and stored in 95 % ethanol until arrival at the laboratory (Table 3). The identification of tick species was performed using the keys and descriptions in Walker *et al.* [24]. After identification, ticks were preserved at −20 °C until DNA extraction. Retrieved ticks belonged to the following species: *Haemaphysalis leachi*, *Rhipicephalus praetextatus*, *R. sanguineus* sensu lato, *R.* aff. *turanicus* [25] and *Amblyomma variegatum*. Of these, a total of 312 ticks were selected from 52 dogs for molecular detection of tick-borne pathogens: 253 adult *H. leachi,* 31 adult *R. praetextatus* and 28 *Rhipicephalus* spp. nymphs. These ticks were grouped in 58 pools according to species and tested for the presence of DNA from *Rickettsia* spp., Anaplasmataceae, *Bartonella* spp. and *Babesia* spp. In addition, 37 fleas were retrieved from 20 dogs and identified based on their morphological characteristics according to the systematic manual of Beaucournu and Launay [26].

### Laboratory methods

#### Serological analysis

Sera were analyzed by two different techniques. Indirect immunofluorescence assay (IFA) was applied using commercial kits to detect the presence of antibodies against *R. conorii* (*Rickettsia conorii* IFA IgG Antibody kit, Fuller Laboratories, Fullerton, CA, USA) and *E. canis* (*Ehrlichia canis* IFA IgG Antibody kit, Fuller Laboratories, Fullerton, CA, USA) as described by the manufacturer. The serum samples were screened at a 1:80 or 1:50 dilution in a phosphate-buffered saline (pH 7.2) for *R. conorii* and *E. canis* assays, respectively. FITC rabbit anti-canine immunoglobulin G conjugates were used as the secondary antibodies. Reactive antibodies were then detected using a fluorescence light microscope (DM LS2, Leica Microsystems, Wetzlar, Germany) at a wavelength of 490 nm. Antibodies against *R. conorii* were also examined by indirect enzyme immunoassay using the Canine *R. conorii* EIA IgG Antibody Kit (Fuller Laboratories, Fullerton, CA, USA) according to the manufacturer’s instructions. Dog sera were diluted 1:100 and incubated in the coated microwells to allow binding of serum antibody to the solid-phase antigens (*R. conorii* outer membrane protein rOmpB). The microwells were then washed to remove unreacted serum proteins and a peroxidase - labelled anti-canine IgG was added to label the bound antibody. After 30 min of incubation at room temperature, the microwells were washed to remove unbound conjugate. The enzyme substrate tetramethylbenzidine (TMB) was then added to quantitate the bound peroxidase activity of the conjugate. After the addition of a stop solution, the absorbance was measured at a wavelength of 450 nm on a microtiter plate reader (Mod 680, Biorad, Hercules, CA, USA).

#### Molecular detection

##### DNA extraction from FTA cards

From each FTA Card, the genomic DNA was extracted following the manufacturer’s instructions with minor modifications. Three punches of each FTA Card measuring 1.2-mm in diameter were used. Punches were washed three times with 100 μl of FTA Purification Reagent, followed by two washing steps with 100 μl of TE-1 Buffer (10 mM Tris–HCl, 0.1 mM EDTA, pH 8.0) and incubated for three minutes at room temperature. Discs were left at room temperature and then used directly as a template in PCR. To ensure that the extraction protocol from ticks and FTA™ Cards was appropriate and could be used in the PCR amplification for hemoparasites, the eukaryotic 18S RNA Pre-Developed TaqMan Assay Reagents (AB, Life Technologies) were used, demonstrating that a negative result corresponded to truly negative samples rather than to a problem with the DNA extraction, sample degradation or PCR inhibition.

##### DNA extraction from ticks

For DNA extraction, ticks were washed with PBS and left overnight in PBS at 4 °C to eliminate ethanol. The DNA was isolated from tick pools by using the High Pure PCR template preparation kit (Roche, Mannheim, Germany) according to the manufacturer’s instructions with some modifications from Solano-Gallego *et al.* [27]. The samples were collected in 2 mL sterile microtubes containing 10 sterile microbeads of 1 mm diameter and 1 microbead of 4 mm diameter and 200 μL of tissue lysis buffer. The tubes were shaken with a TissueLyser (Qiagen) for 2 cycles of 1 min 30 s at a frequency of 25 [28] and incubated overnight at 65 °C with 40 μl of proteinase K.

##### Real time PCR

Real time PCR of *Rickettsia* spp., Anaplasmataceae, *Bartonella* spp. and *Babesia* spp., were carried out in a final volume of 20 μl using FastStart Universal SYBR Green Master (Roche), 4 μl of diluted DNA (1/10 for ticks and 1/2 for blood from FTA Cards) and a final primer concentration depending on the pathogen amplified (Table 1). The thermal cycling profile was 50 °C 2 min and 95 °C 10 min followed by 40 cycles of 95 °C 15 s and 60 °C 1 min and a dissociation curve at the end of the run to assess PCR specificity. The targets amplified for each pathogen and the primers used are shown in Table 1. Water (Water Molecular Biology Reagent®, Sigma) was used as a PCR negative control and positive controls were obtained from commercial slides coated with cells infected with the pathogens or commercial DNA (MegaScreen® FLUOEHRLICHIA c.**,** MegaScreen® FLUOBABESIA canis, MegaScreen® FLUORICKETTSIA ri., MegaScreen® BARTONELLA h. from Megacor). A nested PCR was performed with the samples that gave a positive result for Anaplasmataceae and the product of this PCR was sequenced. A subset of seven samples positive for *Rickettsia* spp. were further characterized by conventional PCR, amplifying several target genes using the primers described in Fernández de Mera *et al.* [29].

**Table 1:** Pathogens and their corresponding probe sequences used to detect pathogen DNA

	Region amplified	Foward primer (5′-3′)	Reverse primer (5′-3′)	Reference	Final [primer] (μM)
Anaplasmataceae	16S rRNA	GCAAGCYTAACACATGCAAGTCG	CTACTAGGTAGATTCCTAYGCATTACTCACC	In house	0.5
Piroplasmida	18S rRNA	GACGATCAGATACCGTCGTAGTCC	CAGAACCCAAAGACTTTGATTTCTCTC	In house	0.3
*Rickettsia* sp.	ITS2	GCTCGATTGRTTTACTTTGCTGTGAG	CATGCTATAACCACCAAGCTAGCAATAC	In house	0.5/0.3
*Bartonella* sp.	ITS1	AGATGATGATCCCAAGCCTTCTG	CCTCCGACCTCACGCTTATCA	Modified from Maggi *et al.* and Gil *et al.* [44, 45]	0.3
Primers used for sequencing					
*Ehrlichia* sp.		GGAATCTACCTAGTAGTACGGAATAGCYA	GTAGGTACCGTCATTATCTTCCCYAY	In house	
*Rickettsia* sp.		RACGGACTAATTRGRGCT	CATTATCTTCCYTGCTAAAAGAG	In house	

##### Sequencing

For species identification, positive samples were characterized at the species level by sequencing the product of the real-time PCR with the exception of *Babesia* spp., which was directly sequenced when possible (*i.e.*, sufficient parasitemia present). Sequences were performed with the BigDye Terminator Cycle Sequencing Ready Reaction Kit (AB, Life Technologies) following the manufacturer’s instructions using the same primers. A region of the 18S rRNA-piroplasmid was amplified for *Babesia* identification [30]. Sequences obtained were compared with those in the GenBank database (www.ncbi.nlm.nih.gov/BLAST).

The new *Rickettsia* sequences were submitted to the EMBL database under accession numbers LM999913-LM999916 and to the GenBank under accession numbers KR181962-KR181977.

#### Morphological blood analysis

Air-dried and stained smears (Diff-Quick®, QCA S.A.) from 43 dogs were examined thoroughly by light microscopy (Nikon Eclipse 50i®). Erythrocyte structure was examined to evaluate cell morphology and shape as well as the presence of abnormal cells, nucleated erythrocytes and erythrocyte agglutination. Leukocyte and platelet counts were estimated according to procedures previously described in the literature [31]. Differential leukocyte count was done by identifying 200 consecutive leukocytes. Morphology and abnormal changes for these two cell lines were also studied. Blood films were examined to check for the presence of infectious agents and intracellular inclusions.

#### Statistical analyses

Age and sex-related differences in dog seroprevalence and differences in the pathogen prevalence in dogs and ticks depending on the study area were tested using Fisher’s exact tests or Chi-square tests using R (R Development Core Team, 2012).

## Results

### Serology

All dogs but one were seropositive to *Rickettsia* spp. antibodies (99.1 %; 95 % Confidence Intervals = 94.8 %-99.9 %) and 29.5 % (95 % C.I. = 21 %-39.2 %) were seropositive to *Ehrlichia* spp. antibodies (Tables 2 and 3). No statistically significant differences in *Rickettsia* spp. seroprevalence between areas were found (in all cases, Fisher’s *p* > 0.05). However, *Ehrlichia* spp. antibodies were more frequently detected in dogs in BI (44.6 %, 95 % C.I. = 31.3 %-58.5 %) than in QE (14.8 %, 95 % C.I. = 4.2 %-33.7 %; Fisher’s *p* = 0.007) and MG (9.1 %, 95 % C.I. = 1.1 %-29.2 %; Fisher’s *p* = 0.003). No age or sex-related differences were detected in seroprevalences.

**Table 2:** Seroprevalence, prevalence of infection, and prevalence of tick infection with *Rickettsia* spp. in rural dogs, Uganda, 2011

	Serology							Molecular detection					
	ELISA			IFA		Total		Blood in FTA			Ticks		
	n^a^	%	95 % C.I.^b^	%	95 % C.I.	%	95 % C.I.	n	%	95 % C.I.	n	%	95 % C.I.
Area													
QE	27	100	81.6-100	92.5	75.7-99.1	100	81.6-100	12	0	0-36.0	21	9.5	1.2-30.4
BI	56	100	90.6-100	100,00	90.6-100	100	90.6-100	20	0	0-23.8	28	21.4	8.3-40.9
MG	22	90.9	70.8-98.9	54.5	32.2-75.6	95	77.1-99.8	6	0	0-57.8	9	33.3	7.5-70.1
Total	105	98.1	93.3-99.8	88.6	80.9-93.9	99.1	94.8-99.9	38	0	0-13.5	58	18.9	9.9-31.4
Age													
Adult	91	97.8	92.3-99.7	91.2	83.4-96.1	98.9	94-100	33	0	0-15.3	49	22.45	11.8-36.6
Young	14	100	68.1-100	71.4	41.9-91.6	100	68.1-100	5	0	0-64.1	9	0	0-44.5
Sex													
Female	41	100	87.4-100	82.9	67.9-92.8	100	87.4-100	11	0	0-38.5	26	19.2	6.6-39-4
Male	64	96.9	89.2-99.6	92.1	82.7-97.4	98.4	91.6-99.9	27	0	0-18.3	32	18.8	7.2-36.4

^a^Number of tick pools

^b^C.I. = Confidence intervals (lower-upper)

**Table 3:** Seroprevalence, prevalence of infection, and prevalence of tick infection with Anaplasmataceae in rural dogs, Uganda, 2011

	Serology (IFA)			Molecular detection					
				Blood in FTA			Ticks		
	n	%	95 % C.I.^a^	n	%	95 % C.I.	n^b^	%	95 % C.I.
QE	27	14.8	4.2-33.7	12	0	0-36	21	19.1	5.5-41.9
BI	56	44.6	31.3-58-5	20	0	0-23.8	28	17.9	6.1-36.9
MG	22	9.1	1.1-29.2	6	0	0-57.8	9	22.2	2.8-60
Total	105	29.5	21-39.2	38	0	0-13.5	58	18.9	9.9-31.4
Age									
Adult	91	28.6	19.6-38.9	33	0	0-15.3	49	14.3	5.9-27.2
Young	14	35.7	12.8-64.9	5	0	0-64.1	9	44.4	13.7-78.8
Sex									
Female	41	36.6	22.1-53.1	11	0	0-38.5	26	19.2	6.5-39.3
Male	64	25	15-37.4	27	0	0-18.3	32	18.8	7.2-36.4

^a^C.I. = Confidence intervals (lower-upper)

^b^Number of tick pools

### Molecular detection in dogs

From the 38 blood samples preserved on FTA cards, three dogs were infected with *B. rossi* (7.8 %, 95 % Confidence Intervals = 1.6 %-21.4 %): two in BI (10 %, 95% CI = 1.2 %-31.7 %) and one in QE (8.3 %, 95% CI = 0.2 %-38.5), without significant differences in prevalence between study areas, or sex and age groups. All blood samples were negative for *Rickettsia* spp.*,* Anaplasmataceae and *Bartonella* spp.

### Smear analysis

No relevant pathological changes were observed in the blood smears. Mild anemia (11.6 % of the dogs), thrombocytopenia (11.6 %), and rouleaux (9.3 %) were the most significant findings in the analyzed smears. Intraerythrocytic piroplasms morphologically compatible with large babesiae were observed in one dog, whereas *Hepatozoon* gamontes were found in a second one.

### Tick and flea infestation

Overall, 40.2 % (95 % CI = 34.4 %-46.9 %) of the examined dogs were parasitized by ticks. We did not retrieve all the ticks observed in the field due to practical limitations, so no data on tick abundance can be provided. Nevertheless, *H. leachi* was the most prevalent species in all of the study areas, representing almost 70 % of the retrieved ticks (Table 4). Fleas were identified as *Ctenocephalides felis* (76 % of the fleas), *Echidnophaga gallinacea* (16 %) and *Pulex irritans* (11 %). No prevalence data is provided because dogs were not systematically searched for fleas.

**Table 4:** Ticks species retrieved from rural dogs, Uganda, 2011

Tick species	Number of ticks	Number of infested dogs	Prevalence (%)	95 % confidence interval (%)
*Haemaphysalis leachi*	324	70	69.3	59.3-78.1
Nymphs of *H. leachi*	1	1	0.9	0-5.4
*Rhipicephalus* spp.:				
*R. praetextatus*	40	14	13.8	7.8-22.2
*R. sanguineus*	4	4	3.9	1.1-9.8
*R. turanicus*	4	4	3.9	1.1-9.8
Nymphs of *Rhipicephalus* sp.	54	32	31.7	22.8-41.7
Larvae of *Rhipicephalus* sp.	2	2	1.9	0.2-6.9
*Amblyomma variegatum*	1	1	0.9	0-5.4
Total	430	101		

### Prevalence of pathogens in ticks

DNA of *Rickettsia* spp. was detected in 18.9 % (95 % C.I. = 9.9 %-31.4 %) of the analyzed tick pools, including ten pools of *H. leachi* and one pool of *R. praetextatus*. Of the seven cases further characterized, two were confirmed as *R. conorii conorii*, and two as *R. massiliae*. Similarly, DNA of Anaplasmataceae was detected in 18.9 % (95 % C.I. = 9.9 %-31.4 %) of tick pools, including ten pools of *H. leachi* and one pool of *R. praetextatus*. One of these cases showed 99 % identity with *E. chaffeensis* (GenBank: CP007480.1) and one showed 99 % with *Anaplasma platys* (GenBank JX112780.1). *Babesia rossi* was detected in one pool of *H. leachi* (1.7 %, 95 % C.I. = 0.0 %-9.2 %) (Tables 2, 3 and 5). Unfortunately, no blood samples were obtained from this dog to determine its infection status, though piroplasms morphologically compatible with *Babesia* spp. were observed in its blood smear. *Bartonella* spp. DNA not detected in any tick pools. Three tick pools were co-infected: two with Anaplasmataceae and *Rickettsia* spp. and one with Anaplasmataceae and *B. rossi*. All *Rhipicephalus* spp. pools were negative.

**Table 5:** Prevalence of tick pathogen infection for study area and tick species, Uganda, 2011

Tick species	Study area											
	Bwindi			Maghinga Gorilla			Queen Elizabeth			Total		
	Pos^a^/Tested	%	95 % C.I.^b^	Pos/Tested	%	95 % C.I.	Pos/Tested	%	95 % C.I.	Pos/Tested	%	95 % C.I.
*Haemaphysalis leachi*												
*Ehrlichia* sp.	5/16	31.3	11-58.6	2/9	22.2	2.8-60	3/15	20.0	4.3-48.10	10/40	25.0	12.7-41.2
*Rickettsia* sp.	5/16	31.3		3/9	33.3	7.5-70.1	2/15	13.3	1.7-40.5	10/40	25.0	12.7-41.2
*Babesia rossii*	0/16	0.0	0-28.7	1/9	11.1	0.3-48.3	0	0	0-30.2	1/40	2.5	0.1-13.2
*Bartonella* sp.	0/16	0.0		0	0.0	0-44.5	0	0	0-20.2	0/40	0	0-12.9
*Rhipicephalus praetextatus*												
*Ehrlichia* sp.	0/5	0.0	0-64.1				1/1	100.0	1.3-100	1/6	16.7	0.4-64.1
*Rickettsia* sp.	1/5	20.0	0.5-71.6				0/1	0.0	0-98.4	1/6	16.7	0.4-64.1
*Babesia rossii*	0/5	0.0	0-64.1				0/1	0.0	0-98.4	0/6	0	0-57.9
*Bartonella* sp.	0/5	0.0	0-64.1				0/1	0.0	0-98.4	0/6	0	0-57.9
*Rhipicephalus* sp. nymph												
*Ehrlichia* sp.	0/7	0.0	0-52.7				0/5	0	0-64.1	0/12	0	0-36
*Rickettsia* sp.	0/7	0.0	0-52.7				0/5	0	0-64.1	0/12	0	0-36
*Babesia rossii*	0/7	0.0	0-52.7				0/5	0	0-64.1	0/12	0	0-36
*Bartonella* sp.	0/7	0.0	0-52.7				0/5	0	0-64.1	0/12	0	0-36
Total	11/28			6/9			6/21			23/58		

^a^Pos = positive

^b^C.I. = Confidence intervals (lower-upper)

No statistically significant differences in the prevalence of pathogens in ticks were observed between study areas and no differences in pathogen prevalence were detected between *H. leachi* and *R. praetextatus* pools.

## Discussion

In the present survey, we show that rural dogs in Uganda are widely exposed to some tick-borne pathogens. We also demonstrate the presence of DNA from important human and animal disease agents in both dogs and associated ticks. We provide molecular evidence of the presence of *Rickettsia* spp. (including the zoonotic *R. conorii conorii* and *R. massiliae)*, Anaplasmataceae (including *E. chaffeensis* and *Anaplasma platys*), and *B rossi.* As far as we know, this study constitutes the first report of *E. chaffeensis* and *B. rossi* in dogs from Uganda or elsewhere in East Africa.

We found that almost all the analyzed dogs were seropositive to *Rickettsia* spp. antibodies. To the best of our knowledge, this is the highest seroprevalence to this pathogen reported in a rural dog population. We are not aware of other similar studies in African dogs. In Spain, a high seroprevalence of 82 % was also observed [32]. In humans, Ndip *et al.* [33] reported *R. africae* antibodies in 26.9 % of the studied population in Cameroon using an IFA. In addition, we found that nearly one in every five tick pools were infected by this agent, also representing a higher prevalence than that reported in similar studies throughout Africa. For example, Socolovschi *et al.* [11] detected only one positive case out of 57 analyzed ticks from dogs in Kampala. Parola *et al.* [16] detected Rickettsial DNA in 7.2 % of ticks from dogs examined in Niger, Mali, Burundi and Sudan, whereas Kamani *et al.* [19] reported a prevalence of 10.5 % in ticks from dogs in Nigeria.

Near one third of the dogs included in the present study were seropositive to *Ehrlichia* spp*.* antibodies. This seroprevalence is higher than that detected in Maasai Mara, Kenya (15.5 % by IFA; [34]) but lower than that reported by Woodroffe *et al.* [35], who detected a seroprevalence of 86 % by IFA in rural dogs in northern Kenya. Reasons for the higher detected seroprevalence in BI are unknown and require further research. We also detected that 18.9 % of the analyzed tick pools were positive to *Ehrlichia* spp. We were able to confirm that at least one of the pools corresponded to *E. chaffeensis* and, to our knowledge, this is the first detection of this zoonotic bacterium in Uganda. A study in Cameroon detected a prevalence of 56 % of *E. chaffeensis* in 63 ticks collected from five dogs from one kennel [15]. Previous studies have shown that *Ehrlichia* species probable emerging human pathogens in sub-Saharan Africa (reviewed in [36]). The detection for the first time of *E. chaffeensis* in Uganda has important implications in public health in this country.

*Babesia rossi* was identified in three dogs and in one tick pool in our survey. The prevalence in our study was nevertheless lower than that in a study carried out in Nigeria by Matjila *et al.* [37], where 65 % of the pools were infected and the majority of *Babesia*-infected dogs (41 %) were only infested with *H. leachi.* Similarly, in our study, as much as 69 % of the dogs were parasitized by this tick species. *Babesia rossi* is considered a natural parasite of indigenous African canids in South Africa [38, 39] and is known to be the most pathogenic for dogs among the three subspecies of *B. canis*, frequently causing a fatal infection despite intensive treatment [38]. It has also been noted that *Babesia* infection can have devastating effects in populations of wild carnivores [40].

No *Bartonella* infections were confirmed in dogs or ticks in our study. In contrast, infection with *Bartonella* spp. has been described in different mammals in Nigeria, Zimbabwe and Kenya [19, 20]. However, it has to be considered that *Bartonella* spp. are difficult to detect in blood due to a low concentration of bacteria [41], and it has been recommended to culture blood before molecular probes, technique that increases *Bartonella* detection in dog blood [42].

## Conclusions

This study confirms that previously undetected vector-borne pathogens of humans and animals are present in Uganda. Detection and identification of zoonotic pathogens is useful for improving diagnosis and applying more specific treatments, and the dog can be a useful sentinel in this regard. Our study also confirmed the importance of analyzing ticks to determine the distribution of tick-borne pathogens in the canine population in Uganda. The interaction of dogs with wildlife and the role they play in the transmission of disease is well known [35, 43]. When dogs live in close association with wildlife (in or near national parks, as in this study) it is imperative to advise dog owners to protect their animals against ectoparasites to prevent the transmission of pathogens such as *B. rossi* to protected carnivores. We strongly recommend the continuation of the monitoring of the studied pathogens in Uganda due to their importance in human, dog and wildlife health.
